# The prognostic factors and multiple biomarkers in young patients with colorectal cancer

**DOI:** 10.1038/srep10645

**Published:** 2015-05-27

**Authors:** Mo-Jin Wang, Jie Ping, Yuan Li, Gunnar Adell, Gunnar Arbman, Bjorn Nodin, Wen-Jian Meng, Hong Zhang, Yong-Yang Yu, Cun Wang, Lie Yang, Zong-Guang Zhou, Xiao-Feng Sun

**Affiliations:** 1 Department of Gastrointestinal Surgery, Institute of Digestive Surgery and State key Laboratory of Biotherapy, West China Hospital, Sichuan University, Chengdu, Sichuan 610041, China; 2 Department of Oncology, and Department of Clinical and Experimental Medicine, Linköping University, Linköping, SE 58183, Sweden; 3 Department of Paediatric Surgery, Institute of Digestive Surgery and State key Laboratory of Biotherapy, West China Hospital, Sichuan University, Chengdu, Sichuan 610041, China; 4 Department of Surgery, and Department of Clinical and Experimental Medicine, Linköping University, Norrköping, SE 60174, Sweden; 5 Department of pathology, Lund University, Lund, SE 22100, Sweden; 6 School of Medicine, Örebro University, Örebro, SE 70182, Sweden

## Abstract

The incidence of colorectal cancer (CRC) in young patients (≤50 years of age) appears to be increasing. However, their clinicopathological characteristics and survival are controversial. Likewise, the biomarkers are unclear. We used the West China (2008-2013, China), Surveillance, Epidemiology, and End Results program (1973-2011, United States) and Linköping Cancer (1972-2009, Sweden) databases to analyse clinicopathological characteristics, survival and multiple biomarkers of young CRC patients. A total of 509,934 CRC patients were included from the three databases. The young CRC patients tended to have more distal location tumours, fewer tumour numbers, later stage, more mucinous carcinoma and poorer differentiation. The cancer-specific survival (CSS) of young patients was significantly better. The PRL (HR = 12.341, 95% CI = 1.615-94.276, P = 0.010), RBM3 (HR = 0.093, 95% CI = 0.012-0.712, P = 0.018), Wrap53 (HR = 1.952, 95% CI = 0.452-6.342, P = 0.031), p53 (HR = 5.549, 95% CI = 1.176-26.178, P = 0.045) and DNA status (HR = 17.602, 95% CI = 2.551-121.448, P = 0.001) were associated with CSS of the young patients. In conclusion, this study suggests that young CRC patients present advanced tumours and more malignant pathological features, while they have a better prognosis. The PRL, RBM3, Wrap53, p53 and DNA status are potential prognostic biomarkers for the young CRC patients.

Colorectal cancer (CRC) is a major cause of cancer mortality worldwide, with an estimated one million new cases and a half million deaths each year^1^. In the United States, although incidence of CRC steadily declined^2^, it is still the third most common cancer and ranked as third leading cause of cancer-related deaths^3^. The same phenomenon was observed in Sweden, where CRC is the second most common cancer type in both men and women^4^. In some Asian countries, such as China, the incidence of CRC has increased 2-4 fold and reached to the level of the Western countries during the past decades^5^.

Besides the improvement of surgical and adjuvant therapy, these decreases of CRC incidence are partially attributed to population based CRC screening which is generally recommended to begin at 50 years of age. In sharp contrast to overall decreasing trends, the incidence of CRC in young patients (≤50 years of age) appears to be increasing^6^. Since one of the earliest articles describing young CRC patients published in 1939^7^, a series of investigations reported the clinicopathological features and survival of young CRC patients. However, because of the likely biases associated with single-institution experiences or limit cohort sizes, the data vary markedly. Most afflicted individuals lack any identifiable risk factor for their development or potential biomarker for prognosis prediction. The mechanisms underlying the apparent increase in CRC among young patients are poorly understood.

In the present study, we analysed clinicopathological characteristics, prognostic factors and survival of young CRC patients from the West China (WC), Surveillance, Epidemiology, and End Results program (SEER) and Linköping Cancer (LC) databases. Furthermore, we assessed the molecular features and the prognostic value of these biomarkers in young CRC patients in LC database.

## Results

### Patient characteristics

We have identified a total of 509,934 eligible patients with CRC in three databases (n = 5,918 in WC, n = 503,002 in SEER and n = 1,014 in LC). Patient demographic and clinicopathological characteristics of each database are shown in Supplementary Table 1. We divided the patients into two groups according to age for analysis: young group (≤50 years of age at diagnosis, n = 43,821) and elderly group (>50 years of age at diagnosis, n = 466,113). There were 530 (9.0%), 43,236 (8.6%) and 55 (5.4%) young patients in WC, SEER and LC, respectively.

### Clinicopathological differences between two age groups

Compared with elderly group, significant differences in young group had been observed concerning the clinicopathological characteristics as follows (Table 1): gender (fewer males in WC and more males in SEER), tumour location (occurring predominately on left colon and the rectum in WC, SEER and LC), tumour numbers (fewer cases with multiple tumours in WC and SEER), TNM stage (later stage in WC and SEER), tumour growth pattern (more frequent in expansive growth in WC), histological type (more mucinous carcinoma in WC and SEER), differentiation (poorer differentiation in WC and SEER), surgical type (more patients underwent radical surgery in WC) and radiotherapy (more patients received radiotherapy in SEER).

### Survival differences between two age groups

**Figure 1:**
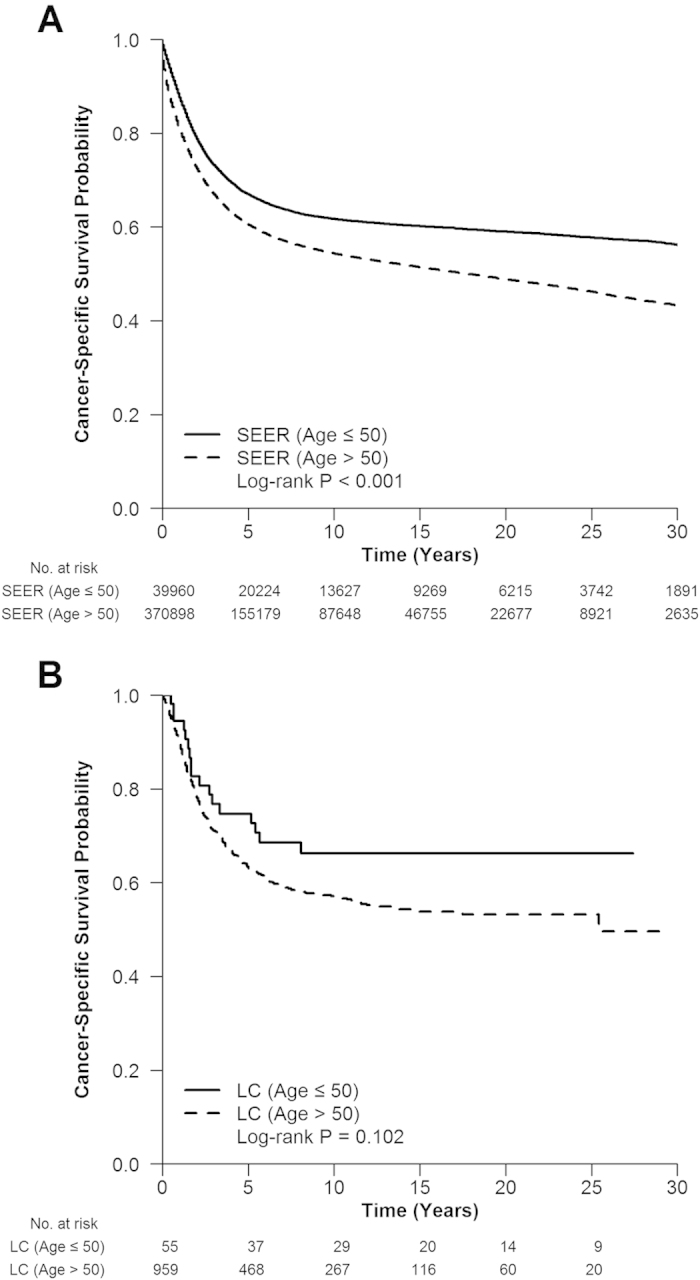
The cancer-specific survival of young and elderly CRC patients in (a) SEER, *P* < 0.001 and (b) LC, *P* = 0.102.

The follow-up information is available in two databases (SEER and LC). The median follow-up period in SEER and LC was 75 months (range, 0-467 months) and 87 months (range, 0-349 months), respectively. In SEER, the 3, 5, 10-year overall cancer-specific survival (CSS) rates were 73.2%, 66.9%, 61.7% in young group, and 66.9%, 60.5%, 54.3% in elderly group, respectively. The CSS of young patients was significantly better than elderly patients (*P* < 0.001, Figure 1a). In LC, the 3, 5, 10-year overall CSS rates were 76.7%, 74.7%, 66.2% in young group, and 71.1%, 62.9%, 56.9% in elderly group, respectively. Similarly, the CSS of young patients was better than elderly patients, although the difference was not statistically significant (*P* = 0.102, Figure 1b). When the survival analyses were stratified by each stage in SEER and LC, the same trend of CSS at stage I (*P* < 0.001, *P* = 0.245, Supplementary Figure 1a), II (*P* < 0.001, *P* = 0.152, Supplementary Figure 1b), III (*P* < 0.001, *P* = 0.524, Supplementary Figure 1c) and IV (*P* < 0.001, *P* = 0.132, Supplementary Figure 1d) had been found.

**Figure 2:**
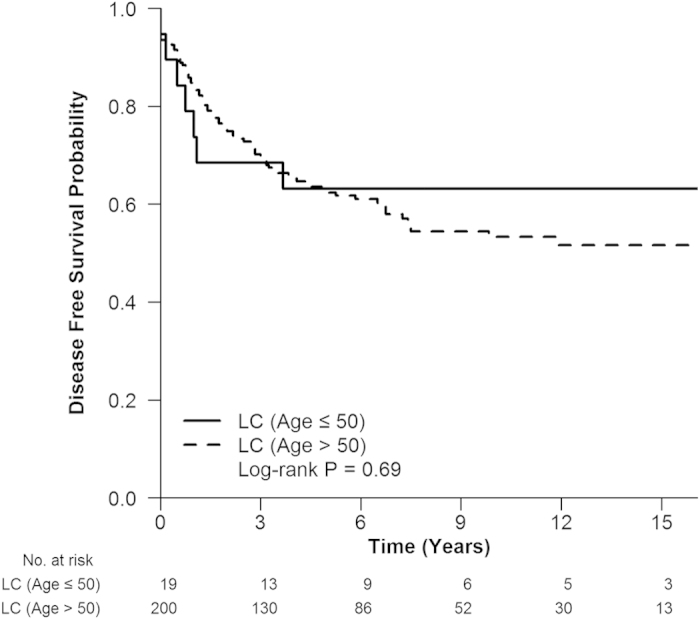
The disease-free survival (DFS) of young and elderly CRC patients in LC. The DFS was not significantly different between two age groups, *P* = 0.690.

In LC, the 3 and 5-year disease-free survival (DFS) rates were 68.4% and 63.2% in young group, and 69.6% and 62.3% in elderly group, respectively. The DFS was not significantly different between two age groups (*P* = 0.690, Fig. 2). Recurrence rate was 27.6% in young group and 34.1% in elderly group (*P* = 0.606). In consideration of recurrence type, local recurrence rate (15.8% *vs*. 13.5%, *P* = 1.000) and distant metastasis rate (26.3% *vs*. 35.0%, *P* = 0.611) had no significant difference between young group and elderly group.

### Multiple biomarkers differences between two age groups

The differences of multiple biomarkers between young and elderly group in LC are shown in Table 2. Compared with elderly group, there were more young patients with moderate/strong PRL (phosphatase of regenerating liver, *P* = 0.014), positive Wrap53 (WD40-encoding RNA antisense to p53, *P* = 0.017), positive RBM3 (RNA-binding motif protein3, *P* = 0.018), weak TAZ (Tafazzin, *P* = 0.044) expression and DNA diploid (*P* = 0.030). The impact of the studied characteristics on prognosis by univariate analyses is presented in Table 3. In young group, TNM stage, tumour growth pattern, surgical type, recurrence, PRL (hazard ratios, HR = 12.341; 95% confidence intervals, CI = 1.615-94.276; *P* = 0.010; Supplementary Figure 2a), RBM3 (HR = 0.093, 95% CI = 0.012-0.712, *P* = 0.018; Supplementary Figure 2b), Wrap53 (HR = 1.952, 95% CI = 0.452-6.342, *P* = 0.031; Supplementary Figure 2c), p53 (HR = 5.549, 95% CI = 1.176-26.178, *P* = 0.045; Supplementary Figure 2d) and DNA status (HR = 17.602, 95% CI = 2.551-121.448, *P* = 0.001; Supplementary Figure 2e) were strongly associated with CSS. Nevertheless, TAZ did not have any prognostic value for CSS although its expression was different in two age groups. Taking into consideration the limited number of young group, we did not further analyse the prognostic value of these biomarkers by multivariable modelling.

### Protein-protein interactions (PPIs) network and pathways of biomarkers in young CRC group

All PPIs for each significant biomarker with a confidence score ≥0.4 (medium confidence) were fetched from the Search Tool for the Retrieval of Interacting Genes/Proteins (STRING) resource (Supplementary Table 2). Then the top 10 confident proteins for each biomarker (total 55 proteins) were used to build the final PPIs network (Supplementary Figure 3) and to do the further gene function enrichment analysis. According to the gene ontcology (GO) enrichment analysis, totally 30 GO terms were enriched with statistically significant raw *P* value and adjusted p value, as shown in Supplementary Table 3, mainly enriched in metabolic process (6 GO terms) and molecular binding functions (8 GO terms, Supplementary Figure 4). With the strict cut-off criterion (adjusted *P* < 0.001), the Kyoto Encyclopaedia of Genes and Genomes (KEGG) pathway enrichment analysis showed a total of four pathways enriched including pathways of Jak-STAT signalling, cell cycle and p53 signalling, as well as pathways in cancer (Supplementary Table 4).

## Discussion

In the present study, we provided large number of CRC patients and extensive clinicopathological data from multiple-institutions in China, U.S. and Sweden. For the first time, the integrated analysis of multiple biomarkers and prognostic factors was performed in young CRC patients compared with elderly patients. Moreover, we utilized the bioinformatics analysis to explore the function of prognostic biomarkers for young CRC patients.

The results described in our study suggested that young CRC patients had distinct clinicopathological characteristics. In accordance with our observations, several investigations showed that CRC in young patients tended to occur predominately on distal location. A literature review of 55 articles concerning young CRC patients exhibited that the sigmoid colon and rectum were the frequent sites (54%)^8^. Similarly, You YN *et al*^9^ identified 64,068 young patients (≤50 years of age) in a large population-based study and found that young-onset CRC commonly arose from the splenic flexure to rectum. Another study based on SEER database also revealed that 32% of CRCs in young patients (35-39 years of age) occurred in the rectum, and these percentages decreased to 15.1% in older patients (>85 years of age)^10^. These findings indicated that the distal colon and rectum were identified as the predilection location of CRC in young patients. It helps us realize that these sites might be the high-yield anatomic regions for endoscopic evaluation in symptomatic young patients and potentially cost-effective targets for screening programs in presymptomatic young adults. In addition, we observed, for the first time, that there were less cases with multiple tumours in young CRC. It is increasingly recognized that a few minor predisposition loci could be responsible for a complex form of CRC heredity^11^. Therefore, such a genetic predisposition could be involved in a part of young CRC patients. Recently, Kirzin S *et al*^12^ found the less frequent synchronous adenoma in sporadic young CRC patients. Taken together, the evidence from this study could reflect accelerated carcinogenesis secondary to predisposing conditions to support this hypothesis.

We showed that young CRC patients had more mucinous carcinoma, poorer differentiation and later stage. A review by O’Connell JB *et al*^8^ indicated that 24% of young CRC patients had mucinous or signet-ring cell carcinomas, 27% were poorly differentiated and 66% presented with later stage. You YN *et al*^9^ also reported that the poor or undifferentiation and advanced-stage were more commonly in young CRC patients. A large-scale study found that 85% of young CRC patients with poorly differentiated tumour presented at stage III or IV, in comparison to only 15% in the elderly patients^13^. The potential reason for young CRC patients with later stage might be lower screening rates and delayed diagnosis. Therefore, some researchers have suggested that average-risk screening begin at younger than 50 years of age^10^.

As with regards to the prognosis of young CRC patients, it remains controversial. In this study, we analysed 43,291 young CRC patients with long follow-up information from two independent databases in U.S. and Sweden. The largest cohort size to date, together with CCS and DFS data, made our results more convincing. Despite these poor prognostic factors likewise have been observed, interestingly, we did find that the 3, 5, 10-year CSS of young patients were significantly better than elderly patients in SEER. The same trend had been seen in LC. The further analysis compared stage-for-stage survival of young with elderly CRC patients. It showed that young patients had better CSS than elderly patients with same stage. Combined with the finding more patients underwent radical surgery and received radiotherapy in young group, our results reflected partially that young patients had a less comorbidities, lower risk of postoperative complications and higher comprehensive treatment completion rate including surgery and adjuvant therapy^8^. In this study, the young group may include hereditary CRC, particularly Lynch syndrome-associated CRC which typically have improved survival compared with non-Lynch syndrome-related CRC. This might be another reason for better survival in young group. Furthermore, we showed that none of DFS, local recurrence and distant metastasis was significantly different between two age groups, which may be relevant to higher proportions of advanced stage disease as well as poorly differentiated and mucinous tumours in young CRC patients. Overall, young CRC patients present advanced tumours occurring distal location and poorer pathological features; nonetheless, these patients had a better prognosis compared with elderly counterparts.

For the first time, we provided an overview of the panel of CRC-related proteins in our published and unpublished data, and performed integrated analysis of more than 25 biomarkers and prognostic factors in CRC patients. Because of the historic association of MSI with Lynch syndrome accounting for 2-7% young CRC patients, most efforts have focused on DNA mismatch repair proteins^14^. Several studies have shown the increased rates of MSI in young CRC patients^15^, whereas Yantiss *et al*^16^ demonstrated the opposite finding in their cohort, with only 4% of patients younger than 40 having MSI tumours compared to 13% of older controls. In our study, we found no significant difference in microsatellite status across age groups, and MSI was not a prognostic biomarker in both young and elderly group. We further found that the PRL, RBM3, Wrap53, TAZ and DNA status were differentially expressed biomarkers between young and elderly group. Additionally, the PRL, RBM3, Wrap53, p53 and DNA status were prognostic biomarkers for young CRC patients. PRL, the gene locating on chromosome 8q24.3, was involved in the metastatic process of CRC. Strong PRL expression could predict resistance to radiotherapy and unfavourable survival in rectal cancer patients with preoperative radiotherapy^17^. Here, we found that PRL expression was negatively related to the CSS of young CRC patients. As a glycine rich protein, high expression of RBM3 has been found to be associated with good prognosis in several types of cancers, including CRC^18^. Consistent with these studies, we showed RBM3 positive expression predicted an improved prognosis in young CRC patients. Wrap53 gene encodes a regulatory RNA essential for p53 function upon DNA damage. The Wrap53 overexpression promotes cellular transformation, whereas Wrap53 knockdown triggers apoptosis of cancer cells. In a previous study, we found increased expression of Wrap53 protein was a predict marker for poor prognosis in CRC patients^19^. Moreover, the p53 positive expression increased with advancing stage and predicted poor survival of CRC patients^20^. Our present study corroborates the finding by Torsello A *et al*^21^ that p53 positive expression was less frequent in young CRC and p53 positivity was an independent predictor of poor survival. Various cancers with increased and abnormal DNA status (DNA non-diploid) have been associated with poor prognosis^22^. In the present study, fewer patients with DNA non-diploid had been observed in young group. DNA non-diploid was strongly associated with poor CSS of both young and elderly CRC patients. It is accordant to our previous study showing similar result in all-aged CRC patients^23^.

These significant biomarkers mainly enriched on metabolic process, molecular binding functions and four signalling pathways including Jak-STAT, cell cycle, p53 and pathways in cancer. The Jak-STAT signalling pathway is the important part of PI3K-Akt signalling pathways which were involved in colorectal carcinogenesis. Accumulating evidence demonstrated that the inhibition of Jak-STAT signalling would lead to cell growth inhibition and induction of apoptosis in CRC cells^24^. Further investigation of these significant biomarkers and their corresponding signalling pathways in a larger population will probably offer the better understanding of the mechanism of underlying cancer development and the prognosis prediction in young CRC patients.

There are several limitations in the present study. Firstly, both the WC and LC databases are collected from a relatively small number of the patients from regional hospitals in China and Sweden, which are not reprehensive of the entire corresponding populations. Although the SEER has been considered a high-quality population-based cancer registry data, it is still limited to 28% of the total U.S. population. Secondly, we are not able to perform a survival analysis in the patients from WC due to a lack of the follow-up data. Thirdly, there is no information on the family history of CRC, therefore we are unable to evaluate the influence of familiar or hereditary CRC particularly Lynch syndrome-associated CRC, if there is, on clinical and biological characteristics. The fourth limitation is that the biomarkers are only examined in a small number of the LC patient samples but not in SEER and WC.

In summary, the present study suggests that young CRC patients have distinct clinicopathological and molecular entity compared to elderly patients. It appears that the young CRC patients tend to occur predominately on distal location, more mucinous carcinoma and poorer differentiation, fewer tumour numbers, and to present later stage. However, the overall CSS of young patients is better than elderly patients. The integrated analysis of more than 25 biomarkers shows that PRL, RBM3, Wrap53, TAZ and DNA status are differentially expressed between young and elderly group. Furthermore, the PRL, RBM3, Wrap53, p53 and DNA status are prognostic biomarkers for young CRC patients. The GO and KEGG pathway enrichment analysis suggests that these significant biomarkers mainly enrich on metabolic process, molecular binding functions and four signalling pathways (Jak-STAT, cell cycle, p53 and pathways in cancer). Our results might provide valuable information for refining the previously debatable description of this specific form of CRC and insight into its precise molecular features.

## Methods

### Database

We respectively used three databases from China, U.S. and Sweden to analyse demographic and clinicopathological characteristics of CRC patients; 1) The WC database included the consecutive patients (2008-2013) from hospitals in the Western China; 2) The SEER database (1973-2011) collected patients from population-based cancer registries in U.S. (based on 2010 census); and 3) The LC database included patients (1972-2009) from the Southeast Swedish Health Care region including hospitals in Linköping, Norrköping, Jönköping, Motala, Eksjö, Varnamo and Vastervik (see supplementary data). The histopathological characteristics, inflammatory infiltration, necrosis and fibrosis were included in this study, according to our published data^25^.

### Biomarker analysis

Immunohistochemistry was performed at our laboratory for the following biomarkers: Astrocyte elevated gene-1 (AEG-1)^26^, CD163^27^, c-erbB-2^28^, cyclooxygenase-2 (Cox-2)^29^, D2-40^30^, FXYD-3^31^, Ki-67^32^, Meningioma associated protein 3 (Mac30)^33^, Nuclear factor-kappaB (NFκB)^34^, p53^20^, p73^35^, Particularly interesting new cysteine-histidine-rich protein (PINCH)^36^, Peroxisome proliferator-activated receptor delta (PPARD)^37^, PRL^17^, ras^38^, RBM3 (unpublished data), TAZ^39^ and Wrap53^19^. The microsatellite status (microsatellite stability, MSS; microsatellite instability, MSI) was determined by PCR based assays as previous describing^40^. Apoptotic cells were detected by the terminal deoxynucleotidy transferase-mediated dUTP-biotin nick end-labelling (TUNEL) assay^41^. DNA content and S-phase fraction (SPF) were measured by flow cytometry. The details were described previously^23^.

### Functional analysis

To further analyse the function of the significant biomarkers, STRING resource was utilized for PPIs network analysis^42^, and the WEB-based Gene Set Analysis Toolkit (WebGestalt) was performed for comprehensive gene functional enrichment analysis^43^, including GO enrichment and KEGG pathway enrichment analysis^44^

### Statistical analysis

The relationships of age groups with clinicopathological characteristics and biomarkers were analysed by Chi-square (χ^2^) test. Survival curves were generated using Kaplan-Meier estimates, differences between the curves were analysed by log-rank test. The impact of each characteristic on survival was examined by the Cox’s proportional hazard regression models. The data were summarized with HR and their 95% CI. The test was two-sided and a *P* value of less than 0.05 was considered statistically significant. All statistical analyses were performed using R software ( http://www.R-project.org/). For details, see supplementary data.

## Additional Information

**How to cite this article**: Wang, M.-J. *et al.* The prognostic factors and multiple biomarkers in young patients with colorectal cancer. *Sci. Rep.* **5**, 10645; doi: 10.1038/srep10645 (2015).

## Supplementary Material

Supplementary Information

## Figures and Tables

**Table 1:** Clinicopathological characteristics of colorectal cancer patients according to age groups.

**Characteristic**	**WC**		***P***	**SEER**		***P***	**LC**		***P***
	**≤50 years n = 530 (%)**	**>50 years n = 5388 (%)**		**≤50 years n = 43,236 (%)**	**>50 years n = 459,766 (%)**		**≤50 years n = 55 (%)**	**>50 years n = 959 (%)**	
Gender									
Male	232 (43.8)	3,333 (61.9)	**<0.001**	22,803 (52.7)	231,126 (50.3)	**<0.001**	24 (43.6)	525 (54.7)	0.108
Female	298 (56.2)	2,055 (38.1)		20,433 (47.3)	228,640 (49.7)		31 (56.4)	434 (45.3)	
Location^a^									
Right Colon	65 (12.3)	1221 (22.7)	**<0.001**	16,021 (38.5)	171,793 (38.8)	**<0.001**	8 (14.5)	292 (30.4)	**0.007**
Left Colon	86 (16.2)	780 (14.5)		13,074 (31.5)	139,756 (31.6)		16 (29.1)	154 (16.1)	
Rectum	379 (71.5)	3387 (62.8)		12,476 (30.0)	130,975 (29.6)		31 (56.4)	513 (53.5)	
Missing	0	0		1,665	17,242		0	0	
Tumour numbers									
Single	185 (96.9)	995 (90.5)	**0.006**	35,423 (81.9)	313,627 (68.2)	**<0.001**	41 (95.3)	747 (94.3)	1.000
Multiple	6 (3.1)	104 (9.5)		7,810 (18.1)	146,091 (31.8)		2 (4.7)	45 (5.7)	
Missing	339	4,289		3	48		12	167	
TNM stage^b^									
I	72 (14.1)	1262 (23.6)	**<0.001**	6,495 (24.0)	70,291 (27.5)	**<0.001**	7 (13.0)	157 (16.8)	0.455
II	115 (22.6)	1741 (32.5)		6,165 (22.8)	75,163 (29.4)		21 (38.9)	345 (37.0)	
III	144 (28.3)	1500 (28.0)		8,058 (29.7)	62,180 (24.3)		14 (25.9)	290 (31.1)	
IV	178 (35.0)	853 (15.9)		6,373 (23.5)	47,931 (18.8)		12 (22.2)	141 (15.1)	
Others^c^	21	32		16,145	204,201		1	26	
Tumour growth pattern									
Expansive	122 (40.8)	599 (28.5)	<**0.001**	/	/		15 (40.5)	356 (49.9)	0.269
Infiltrative	177 (59.1)	1,503 (71.5)		/	/		22 (59.5)	358 (50.1)	
Missing	231	3,286		/	/		18	245	
Histological type									
Non-mucinous	379 (71.5)	4173 (77.4)	**0.002**	36,931 (88.7)	391,999 (90.5)	**<0.001**	49 (90.7)	854 (90.2)	0.893
Mucinous^d^	151 (28.5)	1215 (23.6)		4,694 (11.3)	41,114 (9.5)		5 (9.3)	93 (9.8)	
Others	0	0		1,611	26,653		1	12	
Differentiation^e^									
Well	25 (5.7)	130 (2.7)	**<0.001**	4,112 (13.3)	49,038 (14.8)	**<0.001**	2 (3.7)	60 (6.3)	0.754
Moderately	284 (64.5)	3502 (72.1)		20,013 (64.7)	217,711 (65.8)		36 (66.7)	626 (66.1)	
Poorly+undifferentiated	131 (29.8)	1226 (25.2)		6,811 (22)	64,133 (19.4)		16 (29.6)	261 (27.6)	
Missing	90	530		12,300	128,884		11	2	
Surgical type									
Radical	518 (97.7)	5130 (95.2)	**0.008**	/	/		44 (80.0)	790 (84.8)	0.593
Palliative+unresectable	12 (2.3)	258 (4.8)		/	/		11 (20.0)	164 (17.2)	
Missing	0	0		/	/		0	5	
Radiotherapy									
No	/	/		35,153 (82.2)	412,124 (91.0)	**<0.001**	30 (69.8)	617 (79.1)	0.146
Yes	/	/		7,622 (17.8)	40,814 (9.0)		13 (30.2)	163 (20.9)	
Missing	/	/		461	6,828		12	179	
Chemotherapy									
No	/	/		/	/		42 (95.5)	694 (89.8)	0.221
Yes	/	/		/	/		2 (4.5)	79 (10.2)	
Missing	/	/		/	/		11	186	

^a^Right colon *vs.* distal (left colon+rectum): *P* < 0.001, *P* = 0.007 and *P* = 0.012 in WC, SEER and LC, respectively.

^b^Stage I + II *vs.* III + IV: *P* < 0.001, *P* < 0.001 and *P* = 0.780 in WC, SEER and LC, respectively.

^c^Others include stage 0 and missing cases.

^d^Mucinous carcinoma includes signet-ring cell carcinoma.

^e^Well + Moderately vs poorly+undifferentiated: *P* = 0.037, *P* < 0.001 and *P* = 0.741 in WC, SEER and LC, respectively.

**Table 2:** Analysis of multiple biomarkers in colorectal cancer patients according to age groups.

**Biomarker**	**≤50 years n (%)**	**>50 years n (%)**	***P*****value**
PRL			
Weak	2 (18)	65 (57)	**0.014**
Strong	9 (82)	49 (43)	
Wrap53			
Negative	5 (38)	92 (71)	**0.017**
Positive	8 (62)	38 (29)	
RBM3			
Negative	9 (75)	124 (96)	**0.018**
Positive	3 (25)	5 (4)	
DNA status			
Diploid	10(83)	125 (47)	**0.030**
Non-diploid	2(17)	141 (53)	
TAZ			
Weak	9 (69)	51 (40)	**0.044**
Strong	4 (31)	76 (60)	
D2-40			
Negative	2 (17)	9 (7)	0.244
Positive	10 (83)	117 (93)	
Apoptosis			
<5%	9 (64)	103 (77)	0.272
≥5%	5 (36)	30 (23)	
Fibrosis			
Weak	10 (56)	80 (43)	0.288
Strong	8 (44)	108 (57)	
Microsatellite status			
MSS	14(74)	456 (85)	0.292
MSI	5(26)	79 (15)	
SPF			
<10%	5 (45)	58 (27)	0.308
≥10%	6 (55)	160 (73)	
Cox-2			
Weak	3 (27)	15 (16)	0.328
Strong	8 (72)	81 (84)	
p73			
Weak	3 (25)	46 (39)	0.351
Strong	9 (75)	73 (61)	
PINCH			
<75%	4 (33)	23 (18)	0.389
≥75%	8 (67)	102 (82)	
Necrosis			
<1%	16 (48 )	282 (42)	0.433
≥1%	17 (52)	396 (58)	
CD163			
Weak	8 (73)	105 (82)	0.448
Strong	3 (27)	23 (18)	
Ki-67			
<30%	4 (36)	55 (48)	0.467
≥30%	7 (64)	60 (52)	
FXYD-3			
Weak	4 (33)	56 (44)	0.486
Strong	8 (67)	72 (56)	
PPARD			
Negative	3 (20)	133 (31)	0.518
Positive	12 (80)	292 (69)	
NFKB			
Weak	7 (54)	60 (46)	0.579
Strong	6 (46)	71 (54)	
Mac30			
Weak	3 (30)	50 (41)	0.730
Strong	7 (70)	72 (59)	
p53			
Negative	7 (64)	136 (55)	0.825
Positive	4 (36)	109 (45)	
Inflammatory infiltration			
Weak	9 (50)	97 (52)	0.879
Strong	9 (50)	90 (48)	
AEG-1			
Weak	2 (17)	28 (22)	0.979
Strong	10 (83)	102 (78)	
c-erbB-2			
Weak	5 (42)	113 (40)	1.000
Strong	7 (58)	167 (60)	
ras			
Negative	5 (45)	95 (41)	1.000
Positive	6 (55)	138 (59)	

**Table 3:** Univariate survival analysis of biomarkers and prognostic factors in colorectal cancer patients.

**Variable**	**≤50 years**			**>50 years**		
	**HR**	**95%CI**	***P*****value**	**HR**	**95%CI**	***P*****value**
Gender (Male/Female)	0.460	0.204 - 1.036	0.107	0.918	0.774 - 1.090	0.413
Location (Colon/Rectum)	0.958	0.431 - 2.130	0.930	0.919	0.775 - 1.089	0.416
Tumour numbers (Single/Multiple)	1.765	0.319 - 9.763	0.579	0.744	1.363 - 3.248	0.262
TNM stage (I+II/III+IV)	7.828	2.729 - 22.458	**<0.001**	4.367	3.614 - 5.276	**<0.001**
Tumour growth pattern (Expansive/Infiltrative)	7.889	1.403 - 44.351	**0.020**	1.819	1.486 - 2.227	**<0.001**
Histological type (Non-mucinous/Mucinous^a^)	0.536	0.098 - 2.920	0.534	1.472	1.133 - 1.912	**0.015**
Differentiation (Well+Moderately/Poorly+Undifferentiated)	1.627	0.705 - 3.752	0.335	1.546	1.289 - 1.854	**<0.001**
Surgical type (Radical+Palliative/Unresectable)	5.641	2.394 - 13.289	**<0.001**	6.297	5.211 - 7.608	**<0.001**
Recurrence (No/Yes)	25.327	4.069 - 157.655	**<0.001**	12.71	9.152 - 17.642	**<0.001**
Radiotherapy (No/Yes)	1.444	0.565 - 3.694	0.513	0.884	0.703 - 1.111	0.376
Chemotherapy (No/Yes)	2.27	0.408 - 12.627	0.416	1.793	1.388 - 2.316	**<0.001**
Biomarker						
PRL (Weak/Strong)	12.341	1.615 - 94.276	**0.010**	1.069	0.594 - 1.927	0.850
Wrap53 (Negative/Positive)	1.952	0.452 - 6.342	**0.031**	0.731	0.423 - 1.262	0.342
RBM3 (Negative/Positive)	0.093	0.012 - 0.712	**0.018**	0.995	0.588 - 1.685	0.992
DNA status (Diploid/Non-diploid)	17.602	2.551 - 121.448	**0.001**	1.639	1.212 - 2.216	**0.006**
TAZ (Weak/Strong)	0.508	0.076 - 3.402	0.550	0.651	0.405 - 1.047	0.135
D2-40 (Negative/Positive)	10.974	1.794-18.512	0.464	1.045	0.699 - 1.562	0.061
Apoptosis (<5%/≥5%)	0.529	0.079 - 3.557	0.577	0.418	0.203 - 0.859	**0.040**
Fibrosis (Weak/Strong)	1.387	0.279-7.016	0.739	0.756	0.503 - 1.138	0.260
Microsatellite status (MSS/MSI)	2.058	0.457 - 9.275	0.420	0.825	0.491 - 1.386	0.542
SPF (≤10%/>10%)	4.475	0.596 - 33.614	0.180	0.705	0.419 - 1.188	0.269
Cox-2 (Weak/Strong)	0.207	0.027 - 1.558	0.156	0.716	0.357 - 1.436	0.427
p73 (Weak/Strong)	0.692	0.092 - 5.219	0.763	0.821	0.503 - 1.339	0.504
PINCH (<75%/≥75%)	8.337	0.717-17.484	0.072	0.975	0.584 - 1.627	0.928
Necrosis (<1%/≥1%)	2.918	0.761 - 11.185	0.169	1.238	1.007 - 1.523	0.089
CD163 (Weak/Strong)	1.228	0.163 - 9.26	0.867	0.838	0.428 - 1.643	0.666
Ki67 (<30%/≥30%)	0.341	0.045 - 2.566	0.358	0.696	0.487 - 0.994	0.093
FXYD-3 (Weak/Strong)	1.547	0.391 - 10.044	0.217	1.182	0.728 - 1.922	0.568
PPARD (Negative/Positive)	0.547	0.073 - 4.11	0.618	0.993	0.785 - 1.713	0.162
NFKB (Weak/Strong)	1.079	0.161 - 7.242	0.948	1.951	0.37 - 10.279	0.501
Mac30 (Weak/Strong)	6.245	0.923-10.825	0.246	1.497	0.892 - 2.513	0.198
p53 (Negative/Positive)	5.549	1.176 - 26.178	**0.045**	1.25	0.921 - 1.697	0.227
Infiltration Margin (Weak/Strong)	1.218	0.235 - 6.319	0.843	0.383	0.244 - 0.600	**<0.001**
AEG-1 (Weak/Strong)	7.483	0.730 - 76.719	0.094	2.094	1.090 - 4.020	0.056
c-erbB-2 (Weak/Strong)	4.162	0.685 - 25.301	0.158	0.967	0.727 - 1.287	0.848
ras (Negative/Positive)	1.433	0.344 - 5.966	0.677	1.818	1.299 - 2.544	**0.003**

^a^Mucinous carcinoma includes signet-ring cell carcinoma.
