# Supplementary material for: The prognostic factors and multiple biomarkers in young patients with colorectal cancer
Source: Sci Rep. 2015 May 27;5:10645. doi: 10.1038/srep10645 (PMC4445043; doi:10.1038/srep10645)
Supplement: Supplementary Information [file srep10645-s1.pdf]

The prognostic factors and multiple biomarkers in young patients with colorectal cancer

Mo-Jin Wang [1,2,+], Jie Ping [2,+], Yuan Li [3], Gunnar Adell [2], Gunnar Arbman [4], Bjorn Nodin [5], Wen-Jian Meng [1,2], Hong Zhang [6], Yong-Yang Yu [1], Cun Wang [1], Lie Yang [1], Zong-Guang Zhou [1,*] & Xiao-Feng Sun [1,2,*]

Supplementary methods

Database

We respectively used three databases from China, U.S. and Sweden to analyze demographic and clinicopathological characteristics of CRC patients; 1) The WC database included the consecutive patients (2008-2013) from hospitals in the Western China; 2) The SEER database (1973-2011) collected patients from population-based cancer registries in U.S. (based on 2010 census); and 3) The LC database included patients (1972-2009) from the Southeast Swedish Health Care region including hospitals in Linköping, Norrköping, Jönköping, Motala, Eksjö, Varnamo and Vastervik. The clinicopathological information for each patient was recorded, including the patients' gender, age at diagnosis, tumor location, tumor numbers, TNM stage, growth pattern, histological type and differentiation. All TNM classification was restaged according to the criteria described in the American Joint Committee on Cancer (AJCC) Cancer Staging Manual (7th edition, 2010).

The patients were divided into two groups for analysis: young group (≤50 years of age at diagnosis) and elderly group (>50 years of age at diagnosis). In the SEER database, we included data (November 2013 released) of patients with a diagnosis of primary CRC (C18.0–18.9, 19.9, 20.9 and 260). The SEER histology codes of histology type included: non-mucinous carcinoma (8010, 8140-8141, 8144-8145, 8210-8211, 8220-8221, 8230-8231 and 8260-8263), mucinous carcinoma (8480, 8481 and 8490) and others. Tumor grade was classified as: well differentiated (G1), moderately differentiated (G2), poorly differentiated (G3), and undifferentiated (G4). The cancer-specific survival (CSS) time was calculated from the date of diagnosis to the date of cancer specific death or the end of follow-up (cutoff date: December 2011). Deaths attributed to the cancer of interest were treated as events and deaths from other causes are treated as censored observation. In the LC database, follow-up

was performed by matching all patients against the Swedish Cancer Register and the Cause of Death Register until July 2013. Survival data including local/distant recurrence, disease-free time, survival time and death causes was collected. All patients gave the required informed consent. The surgical specimens once obtained, were snap-frozen immediately in dry ice and stored at -80°C freezer until further biomarker analysis.

Histopathological characteristics analysis

The histopathological characteristics, inflammatory infiltration, necrosis and fibrosis were included in this study, according to our published data. Two investigators including one pathologist independently read the slides in a blinded fashion without any knowledge of the clinicopathological and biological information. The characteristics were determined in 10-20 areas (depending on the size of the section) at x400 magnification. For each tumor/biopsy, 1-5 sections were analyzed, and a mean score was reached. In the cases with discrepant results in the staining score, a consensus score was reached after re-examination[25].

Immunohistochemistry

Immunohistochemistry was performed at our laboratory for the following biomarkers: AEG-1[26], CD163[27], c-erbB-2[28], Cox2[29], D2-40[30], FXYD-3[31], Ki-67[32], Mac30[33], NFκB[34], p53[20], p73[35], PINCH[36], PPARD[37], PRL[17], ras[38], RBM3 (unpublished data), TAZ[39] and Wrap53[19]. Tissue sections were incubated at 60°C for overnight, deparaffinized and hydrated. The activity of endogenous peroxidase was blocked in 3% H_2O_2 in methanol. Nonspecific background staining were immersed in PBS containing 1% BSA and incubated with protein block solution (Spring Bioscience, Pleasanton, CA) for 10 minutes. The sections were then incubated with individual primary antibody and the corresponding secondary antibody. The sections were then rinsed in PBS and incubated for 10 minutes in peroxidase substrate containing 3, 3-diaminobenzidine (DAB) chromogene and counterstained with haematoxylin. In all runs, negative and positive controls were included.

All immunohistochemical slides for each biomarker were independently reviewed scored by two investigators (including one pathologist) without knowledge of clinicopathological and biological information In the case of discrepancy in individual scores, both investigators re-evaluated the slides

together and reached a consensus before combining the individual scores. To avoid an artificial effect, the cells on the margins of the sections and in areas with poor morphology were not counted.

Microsatellite testing and analysis

The microsatellite status (MSS and MSI) was determined using mononucleotide marker BAT-26 as an instability marker, by PCR based assays as previous describing[40]. The BAT-26 locus was amplified by a primary and a secondary PCR using the forward primers: 5′-TGACTACTTTTGACTT CAGCC-3′ and the reverse primer 5′-AACCATTCAACATTTTTAACCC-3′ (Life Technologies, Carlsbad, CA). The primary PCR was carried out in a mixture containing 20 ng DNA, 1 × magnesiumfree buffer, 1.5 mM MgCl2, 0.2 mM dNTP, 2 μM of each primer, 0.5 units Taq polymerase (Promega, Madison, WI)) and water to a final volume of 19.5 μl. The PCR was carried out with initiating denaturation at 94 °C for 4 min, 40 cycles of 94 °C for 1 min, 52 °C for 45 s and 72 °C for 45 s, extension at 72 °C for 10 min. A negative control was included in each run. To incorporate [α-^{33}P]dATP (Amersham Pharmacia Biotech, Bucks, UK) into the samples a secondary PCR was carried out. The secondary PCR was carried out under the same conditions as the primary PCR except that the cycles were reduced to 15. After the PCR the DNA products were denatured by adding 15 μl Blue Juice (containing formamide, xylene cyanol FF, bromophenol blue and EDTA) and incubated at 90 °C for 5 min. The DNA products were separated on a denaturing 6% polyacrylamide gel containing 8 M urea by electrophoresis. The gel was dried and detection was carried out by autoradiography.

TUNEL assay

Apoptotic cells were detected by the terminal deoxynucleotidy transferase-mediated dUTP-biotin nick end-labelling (TUNEL) assay[41]. Five μm-thick sections were cut from the paraffin blocks of the surgical specimen. The sections were deparaffinized in xylene, rehydrated, and incubated with 20 μg/mL proteinase K (Boehringer-Mannheim Biochemicals, Indianapolis, IN) for 15 minutes and rinsed in distilled water. Endogenous peroxidase activity was inhibited with 2% hydrogen peroxide. . The Apop-Tag in situ apoptosis detection kit (Oncor, Gaithersburg, MD) was used to detected apoptosis. The sections were then incubated with equilibration buffer for 10-15 seconds and TdT enzyme in humidified atmosphere at 37 °C for 90 minutes. They subsequently were put into prewarmed working strength stop/wash buffer at room temperature for 10 minutes and incubated with antidigoxigenin-

peroxidase for 45 minutes. Staining was done with 0.05% 3, 3-diaminobenzidine tetrahydrochloride (Sigma Chemical Co. St. Louis, MO), and counter staining was performed in methyl green. A section from rat mammary gland (Oncor) was included in each run as a positive control. To produce DNA fragments, we treated the control sections with 2 μg/mL DNAase at 37 ˚C for 30 minutes before we labeled the control sections by TUNEL assay.

Flow cytometry

DNA content and S-phase fraction (SPF) were measured by flow cytometry. The details were described previously[23]. Fifty μm-thick sections from the paraffin blocks of the surgical specimen were prepared for measurements in a FACScan flow cytometer (Becton-Dickinson, San Jose, CA). Normal diploid cells from the same specimens were used as internal controls. Diploid tumors were defined as having a single G0/1 peak. Tumors were considered non-diploid if there was evidence of more than one distinct G0/1 peak. SPF was estimated using a rectangular model. The number of S-phase cells was calculated by multiplying the number of channels between the G0/1 and G2/M peaks by the mean number of cells per channel in a part of the S-phase interval judged by the operator to be representative. Small disturbing peaks in the S-phase region could be excluded when the SPF, which was divided into two categories: <10% and ≥10%, was calculated. Background correction was made by subtracting a constant estimated in each of the histograms. The mean number of registrations per channel in an area to the right of the G2/M peak was calculated and subtracted from the mean number of registrations per channel in the S-phase interval.

Functional analysis

To further analyze the function of the significant biomarkers, the STRING resource was utilized for PPI network analysis. The corresponding gene symbol of the protein was used for functional analysis. The STRING resource (version 9.1) contained the PPI information from numerous sources including experimental data, computational prediction methods (neighborhood, gene fusion, co-occurrence, and co-expression) and public text collections[42]. All the data from STRING was weighted and integrated for PPI network analysis. A confidence score was assigned to each protein interaction. The score was calculated by benchmarking the performance of the predictions against a common reference set of trusted associations, which was the functional grouping of proteins maintained at

KEGG database. The WebGestalt was utilized for comprehensive gene functional enrichment analysis, including GO enrichment and KEGG pathway enrichment[43, 44]. Human protein coding Entrez genes was set as the reference list for enrichment analysis in the WebGestalt, while hypergeometric distribution algorithm was set as statistical method and Benjamini-Hochberg (BH) method as the multiple test adjustment. A P value of less than 0.05 was considered as the cut-off criterion.

Supplementary Table 1 Demographic and clinicopathological characteristics of colorectal cancer patients

Characteristic	WC (China) n=5,918	SEER (U.S.) n=503,002	LC (Sweden) n=1,014
	n (%)	n (%)	n (%)
Gender			
Male	3,565 (60.2)	253,929 (50.5)	549 (54.1)
Female	2,353 (39.8)	249,073 (49.5)	465 (45.9)
Age (years, Mean ±SD)	64.22 (10.30)	69.34 (14.44)	69.91 (10.46)
Location			
Right colon	1,286 (21.7)	187,816 (38.8)	300 (29.6)
Left colon	866 (14.6)	152,830 (31.6)	170 (16.8)
Rectum	3,766 (63.7)	143,452 (29.6)	544 (53.6)
Missing	0	18,904	0
Tumour numbers			
Single	1,180 (91.5)	349,050 (69.4)	788 (95.5)
Multiple	110 (8.5)	153,901 (30.6)	47 (4.5)
Missing	4,628	51	179
TNM stage			
I	1,334 (22.7)	76,786 (27.2)	164 (16.6)
II	1,856 (31.7)	81,328 (28.8)	366 (37.1)
III	1,644 (28.0)	70,238 (24.8)	304 (30.8)
IV	1,031 (17.6)	54,304 (19.2)	153 (15.5)
Others[a]	53	220,346	27
Tumor growth pattern			
Expansive	721 (30.0)	/	371 (49.4)
Infiltrative	1,680 (70.0)	/	380 (50.6)
Missing	3,517	/	263
Histological type			
Non-mucinous	4,552 (76.9)	428,930 (90.3)	903 (90.2)
Mucinous[b]	1,366 (23.1)	45,808 (9.7)	98 (9.8)
Others	0	28,264	13
Differentiation			
Well	155 (2.9)	53,150 (14.7)	62 (6.2)
Moderately	3,786 (71.5)	237,724 (65.7)	662 (66.1)
Poorly+undifferentiated	1,357 (25.6)	70,944 (19.6)	277 (27.7)
Missing	620	141,184	13

[a] Others include stage 0 and missing cases

[b] Mucinous carcinoma includes signet-ring cell carcinoma

Supplementary Table 2 Top 10 confident proteins of each significant biomarker from PPI analysis by the STRING database

Node1	Node2	Neighborhood	Fusion	Cooccurence	Homology	Coexpression	Experimental	Knowledge	Textmining	Confidence score
TP53	SIRT1	0	0	0	0	0	0.999	0.9	0.935	0.999
	KAT2B	0	0	0	0	0	0.999	0.9	0.902	0.999
	CDKN1A	0	0	0	0	0	0.999	0.9	0.976	0.999
	EP300	0	0	0	0	0	0.999	0.9	0.878	0.999
	ATM	0	0	0	0	0	0.999	0.9	0.976	0.999
	SP1	0	0	0	0	0	0.999	0.9	0.886	0.999
	RCHY1	0	0	0	0	0	0.999	0.9	0.894	0.999
	BRCA1	0	0	0	0	0	0.998	0.9	0.965	0.999
	MDM2	0	0	0	0	0.24	0.999	0.9	0.972	0.999
	USP7	0	0	0	0	0	0.999	0.9	0.889	0.999
TAZ	LCLAT1	0	0	0	0	0	0	0.8	0.499	0.893
	BTRC	0	0	0	0	0	0.846	0	0	0.846
	CCAR1	0	0	0	0	0	0	0	0.811	0.811
	CDH1	0	0	0	0	0	0	0	0.8	0.8
	LATS2	0	0	0	0	0	0	0	0.8	0.8
	AUH	0	0	0	0	0	0	0	0.779	0.778
	DNAJC19	0	0	0	0	0	0	0	0.778	0.778
	YWHAE	0	0	0	0	0	0.741	0	0	0.741
	OPA3	0	0	0	0	0	0	0	0.715	0.714
	TMEM214	0	0	0	0	0	0	0	0.705	0.704
PRL	PRLR	0	0	0	0	0	0.983	0.9	0.979	0.999
	ESR1	0	0	0	0	0	0	0.9	0.953	0.995
	POU1F1	0	0	0	0	0	0	0.9	0.949	0.994
	JAK2	0	0	0	0	0	0	0.9	0.947	0.994

	STAT3	0	0	0	0	0	0	0.9	0.937	0.993
	CALCA	0	0	0	0	0	0	0.9	0.916	0.991
	PTPN11	0	0	0	0	0	0	0.9	0.866	0.985
	ENSG00000235307	0	0	0	0	0	0	0	0.982	0.982
	FSH	0	0	0	0	0	0	0	0.982	0.982
	GHR	0	0	0	0	0	0.351	0.9	0.752	0.981
WRAP53	DKC1	0	0	0	0	0.055	0.665	0.9	0.3	0.973
	TERT	0	0	0	0	0	0	0.9	0.624	0.959
	DDX20	0	0	0	0	0.056	0.621	0	0.279	0.701
	TRNAU1AP	0	0	0	0	0	0.669	0	0.142	0.697
	NCBP2	0	0	0	0	0	0.667	0	0.088	0.676
	KPNB1	0	0	0	0	0	0.621	0	0	0.621
	COIL	0	0	0	0	0	0.621	0	0	0.621
	TGS1	0	0	0	0	0.058	0.58	0	0.14	0.614
	PPP6C	0	0	0	0	0	0.581	0	0	0.581
	SNRPC	0	0	0	0	0	0.387	0	0.108	0.417
RBM3	HNRNPA2B1	0	0	0.522	0.865	0.088	0.621	0	0.305	0.667
	RBMX	0	0	0.525	0.884	0.625	0	0	0.305	0.657
	FTSJ1	0	0	0	0	0.522	0	0	0.23	0.607
	CHAF1A	0	0	0	0	0	0.603	0	0	0.603
	NONO	0	0	0	0.716	0.601	0	0	0.133	0.6
	RBMX2	0	0	0.511	0.759	0.523	0	0	0	0.577
	ATP6AP2	0	0	0	0	0.581	0	0	0	0.571
	RPL10	0	0	0	0	0.555	0	0	0.052	0.555
	UXT	0	0	0	0	0.538	0	0	0.069	0.54
	VBP1	0	0	0	0	0.542	0	0	0.051	0.531

Supplementary Table 3 GO enrichment results of significant biomarkers from the WebGestalt resource

GO ID	GO Name	Count	Ratio of enrichment	raw *P* value	adjusted *P* value
GO:0044238	primary metabolic process	48	1.61	3.40E-09	3.58E-07
GO:0071704	organic substance metabolic process	48	1.56	1.61E-08	6.14E-07
GO:0043231	intracellular membrane-bounded organelle	47	1.62	2.77E-08	1.27E-07
GO:0043170	macromolecule metabolic process	46	1.84	2.27E-10	3.19E-08
GO:0044260	cellular macromolecule metabolic process	45	1.97	5.01E-11	2.11E-08
GO:0005515	protein binding	42	1.69	6.94E-07	2.01E-05
GO:0090304	nucleic acid metabolic process	35	2.29	1.03E-08	6.14E-07
GO:0070013	intracellular organelle lumen	30	2.99	8.57E-10	8.23E-09
GO:0043233	organelle lumen	30	2.95	1.22E-09	9.76E-09
GO:0031974	membrane-enclosed lumen	30	2.91	1.69E-09	1.16E-08
GO:0044428	nuclear part	29	3.1	8.55E-10	8.23E-09
GO:0031981	nuclear lumen	28	3.36	3.37E-10	5.39E-09
GO:0005654	nucleoplasm	26	5.76	9.43E-15	4.53E-13
GO:0051246	regulation of protein metabolic process	20	4.09	1.52E-08	6.14E-07
GO:0044703	multi-organism reproductive process	17	6.83	1.24E-10	2.61E-08
GO:0019899	enzyme binding	15	4.06	1.95E-06	3.77E-05
GO:0044451	nucleoplasm part	15	5.99	1.24E-08	6.61E-08
GO:0042802	identical protein binding	13	4.44	4.18E-06	4.85E-05
GO:0003723	RNA binding	13	4.53	3.36E-06	4.85E-05
GO:0016604	nuclear body	13	15.52	1.30E-12	3.12E-11
GO:0006913	nucleocytoplasmic transport	11	9.41	1.59E-08	6.14E-07
GO:0008134	transcription factor binding	9	6.15	1.27E-05	0.0001
GO:0019904	protein domain specific binding	9	4.89	7.58E-05	0.0005
GO:0000975	regulatory region DNA binding	7	6.14	0.0001	0.0005

GO:0044212	transcription regulatory region DNA binding	7	6.29	0.0001	0.0005
GO:0003682	chromatin binding	7	6.53	8.91E-05	0.0005
GO:0000060	protein import into nucleus, translocation	6	43.27	4.47E-09	3.76E-07
GO:0002039	p53 binding	6	37.49	1.11E-08	6.44E-07
GO:0015030	Cajal body	6	40.89	6.70E-09	4.02E-08
GO:0060397	JAK-STAT cascade involved in growth hormone signaling pathway	5	60.09	1.65E-08	6.14E-07

Supplementary Table 4

KEGG pathway enrichment results of significant biomarkers from the WebGestalt resource.

KEGG ID	Pathway Name	No. of genes	Genes	Ratio of enrichment	raw *P* value	adjusted *P* value
hsa04630	Jak-STAT signaling pathway	7	EP300, PRLR, JAK2, PRL, STAT3, GHR, PTPN11	17.28	1.52E-07	4.86E-06
hsa04110	Cell cycle pathway	6	CDKN1A, EP300, TP53, MDM2, YWHAE, ATM	18.52	8.39E-07	9.86E-06
hsa04115	p53 signaling pathway	5	CDKN1A, TP53, MDM2, RCHY1, ATM	28.14	9.24E-07	9.86E-06
hsa05200	Pathways in cancer	6	CDKN1A, EP300, TP53, MDM2, CDH1, STAT3	7.04	0.0002	0.0002

Supplementary Figure Legends

Supplementary Figure 1. The cancer-specific survival of young and elderly CRC patients with (a) stage I (SEER, $P<0.001$ and LC, $P=0.245$), (b) stage II (SEER, $P<0.001$ and LC, $P=0.152$), (c) stage III (SEER, $P<0.001$ and LC, $P=0.524$) and (d) stage IV (SEER, $P<0.001$ and LC, $P=0.132$).

Supplementary Figure 2. The prognostic value of biomarkers in young and elderly groups (a) PRL ($P=0.010$, $P=0.850$), (b) RBM3 ($P=0.018$, $P=0.992$), (c) Wrap53 ($P=0.031$, $P=0.342$), (d) p53 ($P=0.045$, $P=0.227$) and (e) DNA status ($P=0.001$, $P=0.006$).

Supplementary Figure 3. The PPIs network for significant biomarkers in young CRC patients. The top 10 confident proteins for each biomarker from the STRING database (total 55 proteins) were involved in the analysis.

Supplementary Figure 4. Visualization of significantly enriched GO terms with three separate Directed Acyclic Graphs (DAGs) revealed the hierarchical relationship of enriched GO terms. Each GO term is a node in the DAG. GO terms in red have a $P<0.05$, while the black ones are their non-enriched parents.

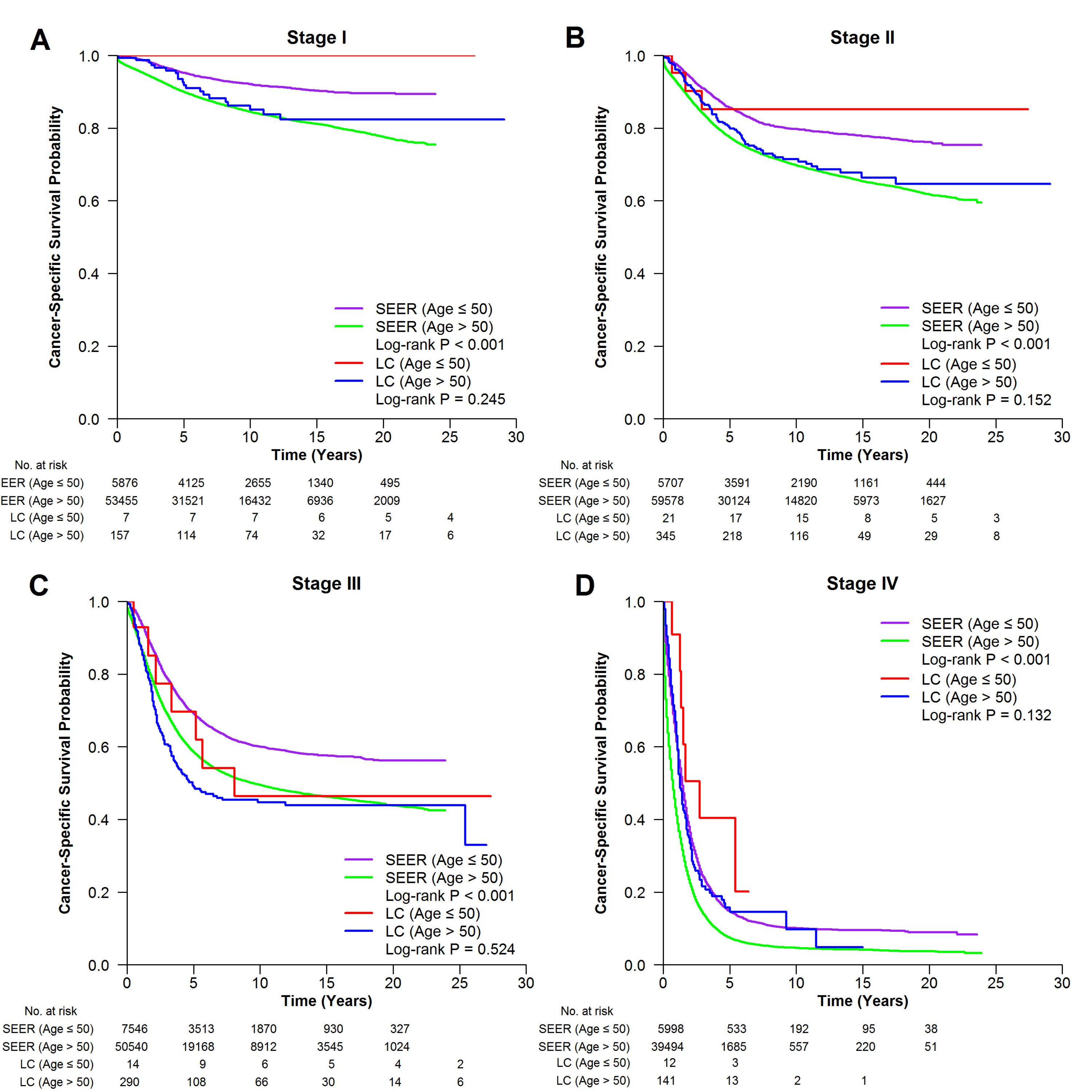
A
Stage I
Cancer-Specific Survival Probability
1.0
0.8
0.6
0.4
0.2
0.0
0
5
10
15
20
25
30
Time (Years)
SEER (Age ≤ 50)
SEER (Age > 50)
Log-rank P < 0.001
LC (Age ≤ 50)
LC (Age > 50)
Log-rank P = 0.245
No. at risk
EER (Age ≤ 50) 5876 4125 2655 1340 495
EER (Age > 50) 53455 31521 16432 6936 2009
LC (Age ≤ 50) 7 7 7 6 5 4
LC (Age > 50) 157 114 74 32 17 6
B
Stage II
Cancer-Specific Survival Probability
1.0
0.8
0.6
0.4
0.2
0.0
0
5
10
15
20
25
30
Time (Years)
SEER (Age ≤ 50)
SEER (Age > 50)
Log-rank P < 0.001
LC (Age ≤ 50)
LC (Age > 50)
Log-rank P = 0.152
No. at risk
SEER (Age ≤ 50) 5707 3591 2190 1161 444
SEER (Age > 50) 59578 30124 14820 5973 1627
LC (Age ≤ 50) 21 17 15 8 5 3
LC (Age > 50) 345 218 116 49 29 8
C
Stage III
Cancer-Specific Survival Probability
1.0
0.8
0.6
0.4
0.2
0.0
0
5
10
15
20
25
30
Time (Years)
SEER (Age ≤ 50)
SEER (Age > 50)
Log-rank P < 0.001
LC (Age ≤ 50)
LC (Age > 50)
Log-rank P = 0.524
No. at risk
SEER (Age ≤ 50) 7546 3513 1870 930 327
SEER (Age > 50) 50540 19168 8912 3545 1024
LC (Age ≤ 50) 14 9 6 5 4 2
LC (Age > 50) 290 108 66 30 14 6
D
Stage IV
Cancer-Specific Survival Probability
1.0
0.8
0.6
0.4
0.2
0.0
0
5
10
15
20
25
30
Time (Years)
SEER (Age ≤ 50)
SEER (Age > 50)
Log-rank P < 0.001
LC (Age ≤ 50)
LC (Age > 50)
Log-rank P = 0.132
No. at risk
SEER (Age ≤ 50) 5998 533 192 95 38
SEER (Age > 50) 39494 1685 557 220 51
LC (Age ≤ 50) 12 3
LC (Age > 50) 141 13 2 1

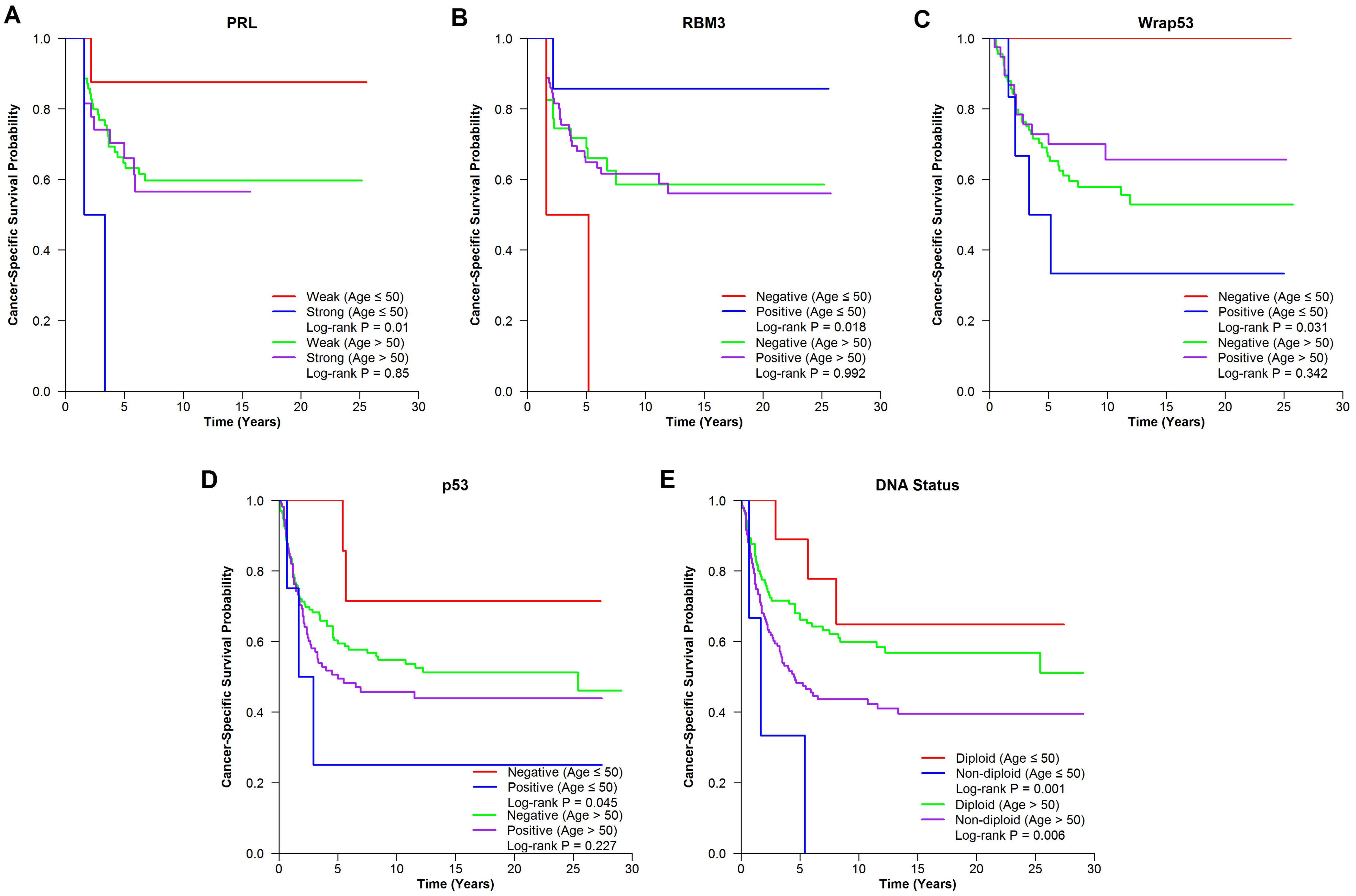
A
PRL
Weak (Age ≤ 50)
Strong (Age ≤ 50)
Log-rank P = 0.01
Weak (Age > 50)
Strong (Age > 50)
Log-rank P = 0.85
B
RBM3
Negative (Age ≤ 50)
Positive (Age ≤ 50)
Log-rank P = 0.018
Negative (Age > 50)
Positive (Age > 50)
Log-rank P = 0.992
C
Wrap53
Negative (Age ≤ 50)
Positive (Age ≤ 50)
Log-rank P = 0.031
Negative (Age > 50)
Positive (Age > 50)
Log-rank P = 0.342
D
p53
Negative (Age ≤ 50)
Positive (Age ≤ 50)
Log-rank P = 0.045
Negative (Age > 50)
Positive (Age > 50)
Log-rank P = 0.227
E
DNA Status
Diploid (Age ≤ 50)
Non-diploid (Age ≤ 50)
Log-rank P = 0.001
Diploid (Age > 50)
Non-diploid (Age > 50)
Log-rank P = 0.006
Cancer-Specific Survival Probability
Time (Years)

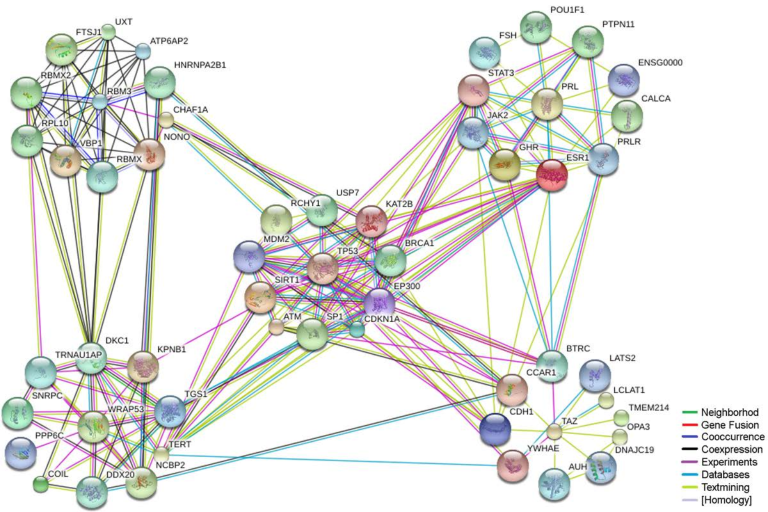

UXT
FTSJ1
ATP6AP2
HNRNPA2B1
RBMX2
RBM3
CHAF1A
RPL10
NONO
VBP1
RBMX
RCHY1
USP7
KAT2B
MDM2
TP53
BRCA1
SIRT1
EP300
ATM
SP1
CDKN1A
DKC1
TRNAU1AP
KPNB1
SNRPC
WRAP53
TGS1
PPP6C
TERT
COIL
NCBP2
DDX20
POU1F1
PTPN11
FSH
ENSG0000
STAT3
PRL
CALCA
JAK2
GHR
PRLR
ESR1
BTRC
LATS2
CCAR1
LCLAT1
TMEM214
CDH1
TAZ
OPA3
YWHAE
DNAJC19
AUH
Neighborhod
Gene Fusion
Cooccurrence
Coexpression
Experiments
Databases
Textmining
[Homology]

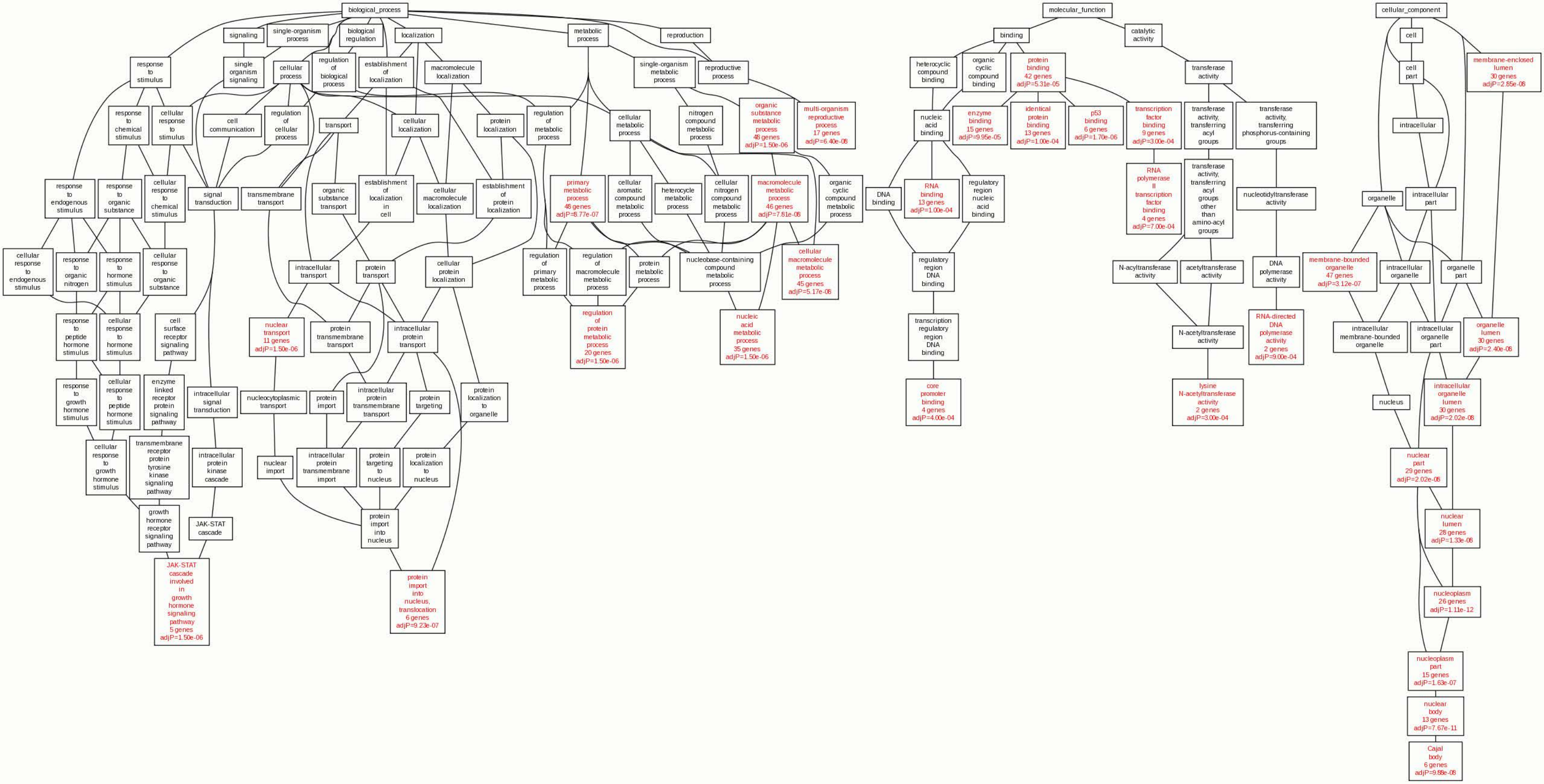
biological_process
signaling
single-organism process
biological regulation
localization
metabolic process
reproduction
response to stimulus
single organism signaling
cellular process
regulation of biological process
establishment of localization
macromolecule localization
single-organism metabolic process
reproductive process
response to chemical stimulus
cellular response to stimulus
cell communication
regulation of cellular process
transport
cellular localization
protein localization
regulation of metabolic process
cellular metabolic process
nitrogen compound metabolic process
organic substance metabolic process 48 genes adjP=1.50e-06
multi-organism reproductive process 17 genes adjP=6.40e-08
response to endogenous stimulus
response to organic substance
cellular response to chemical stimulus
signal transduction
transmembrane transport
organic substance transport
establishment of localization in cell
cellular macromolecule localization
establishment of protein localization
primary metabolic process 48 genes adjP=8.77e-07
cellular aromatic compound metabolic process
heterocycle metabolic process
cellular nitrogen compound metabolic process
macromolecule metabolic process 46 genes adjP=7.81e-08
organic cyclic compound metabolic process
cellular response to endogenous stimulus
response to organic nitrogen
response to hormone stimulus
cellular response to organic substance
intracellular transport
protein transport
cellular protein localization
regulation of primary metabolic process
regulation of macromolecule metabolic process
protein metabolic process
nucleobase-containing compound metabolic process
cellular macromolecule metabolic process 45 genes adjP=5.17e-08
response to peptide hormone stimulus
cellular response to hormone stimulus
cell surface receptor signaling pathway
nuclear transport 11 genes adjP=1.50e-06
protein transmembrane transport
intracellular protein transport
regulation of protein metabolic process 20 genes adjP=1.50e-06
nucleic acid metabolic process 35 genes adjP=1.50e-06
response to growth hormone stimulus
cellular response to peptide hormone stimulus
enzyme linked receptor protein signaling pathway
intracellular signal transduction
nucleocytoplasmic transport
protein import
intracellular protein transmembrane transport
protein targeting
protein localization to organelle
cellular response to growth hormone stimulus
transmembrane receptor protein tyrosine kinase signaling pathway
intracellular protein kinase cascade
nuclear import
intracellular protein transmembrane import
protein targeting to nucleus
protein localization to nucleus
growth hormone receptor signaling pathway
JAK-STAT cascade
protein import into nucleus
JAK-STAT cascade involved in growth hormone signaling pathway 5 genes adjP=1.50e-06
protein import into nucleus, translocation 6 genes adjP=9.23e-07
molecular_function
binding
catalytic activity
heterocyclic compound binding
organic cyclic compound binding
protein binding 42 genes adjP=5.31e-05
transferase activity
nucleic acid binding
enzyme binding 15 genes adjP=9.95e-05
identical protein binding 13 genes adjP=1.00e-04
p53 binding 6 genes adjP=1.70e-06
transcription factor binding 9 genes adjP=3.00e-04
transferase activity, transferring acyl groups
transferase activity, transferring phosphorus-containing groups
DNA binding
RNA binding 13 genes adjP=1.00e-04
regulatory region nucleic acid binding
RNA polymerase II transcription factor binding 4 genes adjP=7.00e-04
transferase activity, transferring acyl groups other than amino-acyl groups
nucleotidyltransferase activity
regulatory region DNA binding
N-acyltransferase activity
acetyltransferase activity
DNA polymerase activity
transcription regulatory region DNA binding
N-acetyltransferase activity
RNA-directed DNA polymerase activity 2 genes adjP=9.00e-04
core promoter binding 4 genes adjP=4.00e-04
lysine N-acetyltransferase activity 2 genes adjP=3.00e-04
cellular_component
cell
cell part
membrane-enclosed lumen 30 genes adjP=2.85e-08
intracellular
organelle
intracellular part
membrane-bounded organelle 47 genes adjP=3.12e-07
intracellular organelle
organelle part
intracellular membrane-bounded organelle
intracellular organelle part
organelle lumen 30 genes adjP=2.40e-08
nucleus
intracellular organelle lumen 30 genes adjP=2.02e-08
nuclear part 29 genes adjP=2.02e-08
nuclear lumen 28 genes adjP=1.33e-08
nucleoplasm 26 genes adjP=1.11e-12
nucleoplasm part 15 genes adjP=1.63e-07
nuclear body 13 genes adjP=7.67e-11
Cajal body 6 genes adjP=9.88e-08
